# Retraction: A photon thermal diode

**DOI:** 10.1038/ncomms16134

**Published:** 2017-08-21

**Authors:** Zhen Chen, Carlaton Wong, Sean Lubner, Shannon Yee, John Miller, Wanyoung Jang, Corey Hardin, Anthony Fong, Javier E. Garay, Chris Dames

Nature Communications 5: Article number: 5446 ; DOI: 10.1038/ncomms6446 (2014); Published: 11 17 2014; Updated: 08 21 2017

Because two of its three major findings have been invalidated, the authors wish to retract this Article^1^. Budaev^2^ correctly identifies a fundamental symmetry error in the way the ‘inelastic thermal collimation’ was configured in several crucial experiments of ref. 1, specifically the results presented in Fig. 3c (the three filled and four striped bars, labeled ‘Col. 1’ and ‘Col. 2’, respectively) and Fig. 4. As detailed in the accompanying Correspondence^3^, this error originated from a faulty thermal estimate^4^, and further modelling^3^,^4^ now confirms the problem. Although we continue to believe the heat flow measurements in ref. 1 were accurate for all of the configurations presented, due to the symmetry error none of those experimental configurations were actually relevant for two of the most important findings of the paper, which are therefore retracted: firstly, that a photon thermal diode was experimentally demonstrated, and secondly, that the ‘inelastic thermal collimation’ mechanism is a suitable nonlinearity for realizing thermal rectification when combined with asymmetric scattering structures (e.g., copper pyramids or etched triangular pores in silicon).

The symmetry error^2^ does not apply to the experiments without thermal collimation, specifically the results presented in Fig. 3c for photons (the six leftmost, unfilled bars) and Supplementary Figure 12 for phonons. Therefore, the last major conclusion of the Article^1^ remains well-supported by those original experiments: Asymmetric scattering alone is insufficient to achieve thermal rectification.
